# Bibliography

**Published:** 1897-12

**Authors:** 


### Bibliography.

A Manual of the Injuries and Surgical Diseases of the Face, Mouth, and Jaws. By John Sayre Marshall, M.D. (Syracuse University), former Professor of Dental Pathology and Oral Surgery and Emeritus Professor of Oral Surgery of the Dental Department of Northwestern University, etc. Philadelphia: S. S. White Dental Manufacturing Company, 1897.

It is by no means a common experience to feel, after the examination of a new book, that it is entirely satisfactory in its special line of work, but this volume, prepared by Dr. Marshall, approaches very nearly this desirable position, and the author deserves special commendation for his very great, and it is believed successful, labor in placing the subject of surgical injuries and diseases of the mouth and jaws so satisfactorily before the dental reading world.

His idea of preparing such a work is explained in his preface, and as this book has been arranged on new lines, and for convenience of teaching, it is proper his reasons should be given for this departure from established custom. "The plan of this volume is the outgrowth of several years' experience as a teacher of oral surgery in medical and dental colleges. During these years the author has been more and more impressed with the disadvantages under which teachers and students have labored in the old system of teaching by didactic lectures. The same feeling has been growing year by year among the teachers in the American medical and dental colleges, and many of them have expressed themselves as anxious to adopt a recitation system of teaching in their special departments. The greatest objection which has been raised to the inauguration of such a system of teaching has been the lack of text-books arranged upon a suitable plan for teaching by this method."

It is certainly true that the methods heretofore adopted in medical and dental teaching have followed very strictly the form of didactic lectures, and any departure from this ancient system of teaching has been frowned upon by the elders as being at once undignified and contrary to established precedent. On the other hand, there has been a growing sentiment among teachers in professional work that the old plan of didactic lectures has become antiquated and necessarily ineffective with the advance of knowledge in all departments, and that, therefore, the objective method should to a large degree supplant it. It may be questioned whether either of these extreme views should be considered as correct. The old system has the positive advantage in permitting a broader view of the subject than would be possible in a mere series of talks with recitations following. This has the merit which the quiz-master possesses of fastening the subject in outline upon the memory. In the opinion of the writer a combination of the two is the proper course. This is apparently what Dr. Marshall proposes, and his method would be eminently successful, providing all teachers made use of his book and followed it closely; otherwise the questions added to each chapter would be of little value.

For the teacher who appreciates his own experience and personal investigation, simply superadding book-information as a supplementary work, this method would be very distasteful, and it will, in all probability, be rejected. It is, therefore, thought that the author's very great additional labor in preparing the aforesaid questions to every chapter might have been dispensed with, but the fact that they are there will be an incentive to many to adopt this plan, which the few have long since introduced as essential to correct teaching.

The author of this work of seven hundred and ten pages, including index, modestly terms it a manual. The name is unfortunate, as it presupposes a similarity to a class of books with which all are familiar, mere compends of the subjects treated. To regard this volume in that light would be a grave error, for while the author does not claim to be exhaustive in his treatment, or even wholly original, he has been able to condense every subject so that nothing seems to be lacking to enable the reader to clearly understand the subject.

The repetition of the names of authorities quoted seems to the writer to mar the pages. The names of Senn and Warren grow somewhat wearisome, especially as the subject-matter quoted is generally not original with these authors; in fact, frequently the recognized property of the profession for a long period. In illustration of this, the following sentence is quoted: "Senn says inflammation is not a disease, but a symptom." This the author might very properly have written himself without being called to account for it, as it is a recognized truism in pathology that the phenomena of inflammation is preceded by a cause, the old idea of idiopathic inflammation having no foundation in fact. While the authority of Senn is recognized wherever his works are known, it is rather surprising to find the following quoted for young men to absorb as part of their mental pabulum: "Senn says, recognizing the fact that acute inflammation, wherever it occurs, is the result of the action of certain specific micro-organisms upon the vessel-walls and the tissues outside of them, the rational treatment would seem to be to destroy the microbes in the tissues as soon as their presence is discovered, by the saturation of the tissues with some solution having germicidal powers." As a germicidal solution would certainly destroy the tissue, it is inconceivable that any one should suggest this as a "rational treatment." If the word inhibit had been used, it might have a decided value, for that is about the extent we can safely go with our antiseptics. The rational treatment in inflammation is to endeavor to remove the cause of these secondary phenomena, if that be possible, which, unfortunately is rarely the case.

The pathology of the book is, in the writer's opinion, of very great value; indeed, we have no work adapted for dental use at all comparable to it, and where oral surgery, so called, is made a part of dental instruction it will become a text-book of first importance. While the writer fails to see the necessity of making a specialty of the surgery of the mouth, as surgery there in nowise differs from surgery anywhere throughout the human organism, it still has become a distinctive part of dental teaching. Hence this book will occupy a place in dental college work not heretofore filled, except by Dr. Garretson's book, which, notwithstanding many defects, must continue to hold a first place in the work which he originated. This is, however, too voluminous for general use: hence this volume is welcomed, and it is hoped the author will avoid the temptation of enlarging it in succeeding editions. It is altogether more satisfactory as it is.

It is not proposed in this review to cover the book in detail. The writer has found repeatedly matter not quite in accord with his ideas, but it would be pure hypercriticism to enlarge upon these in the face of so much good work in other directions. The mere statement of the fact that sixty chapters, covering the entire subject, comprise the contents, will, it is presumed, be sufficient to give an idea of the great labor bestowed upon its production.

It is anticipated that the book will find its way into all dental schools, for it can be recommended as a text-book upon oral surgery without any reservations.

The publishers have prepared this volume with their usual care in regard to type, paper, and general make-up.

The prices are: cloth binding, $6.00; sheep, $7.00.

Anatomy, Descriptive and Surgical. By Henry Gray, F.R.S., Lecturer on Anatomy at St. George's Hospital, London. New and thoroughly revised American edition, much enlarged in text and engravings, both in colors and in black. In one imperial octavo volume of 1239 pages, with seven hundred and seventy-two large and elaborate engravings. Price, with illustrations, colors, cloth, $7.00 ; leather, $8.00. In black cloth, $6.00; leather, $7.00. Philadelphia and New York: Lea Brothers & Co.

It would seem to be a superfluous effort to review a new edition of Gray's Anatomy, so thoroughly has this become identified with the study of the human organism and which for forty years has held, undisputed, a permanent place, but in this new edition, presented by the publishers, there has been given so much that brings the entire work up to the present standard of knowledge, that it comes to us with a renewed confidence that the work will remain the standard for many years to come. The publishers in their note to this edition very truly say, "Anatomy is far from stationary either in facts or improvement in the methods of their presentation; hence any work which would faithfully reflect the existing position of the science must be revised at comparatively frequent intervals."

This work, with index, covers 1249 pages, with seven hundred and seventy-two illustrations, many of which are new, and it would be simply an iteration of a well-known fact to say that the student of anatomy would prefer this book over all others for its broad comprehensive treatment of all the subjects considered. Its greatest claim in the view of the writer has always been its clear elucidation of anatomical problems, bringing each topic treated within the comprehension of the beginner, and at the same time satisfying all the requirements of the most erudite. This is the true mission of all teaching, and books fail, where failure exists, in not meeting this educational demand.

It has been customary in works of this kind to pass over the dental portion in a cursory manner entirely unsatisfactory to the students of dentistry, and it is therefore a gratification to observe that the publishers have, with a recognized liberal spirit, revised and enlarged the chapter on "The Organs of Digestion" by a very full and satisfactory treatment of this portion of the work. This was given in charge of Dr. Burchard, and it adds materially to the value of the book from the dentist's point of observation.

Gray's Anatomy has become so much a part of the educational work of the schools that it would be a superfluous task to enlarge further upon its merits. It is beyond all controversy the one book, above all others, to place in the hands of the student and the one most worthy of continued reference by the practitioners in medicine and the specialties connected therewith.

Zahn und Mundleiden mit Bezug auf Allgemein-Erkrankungen. Ein Wegweiser für Aerzte und Zahnärzte. Von Zahnarzt P. Ritter. Mit 20 Abbildungen. Verlag von Fischers Medicin Buchhandlung. Berlin: H. Kornfeld, 1897.

This book has been prepared by the author to fill a special place in the literature of dentistry, that of diseases of the teeth and oral cavity.

The author begins his work with a statement of the present condition of dentistry. After a brief chapter on the action of the mouth bacteria, he takes up the extraction of teeth, and devotes considerable space, very properly, to antiseptics in extraction, giving his method, which does not materially differ from that in common use in this country by all careful operators.

On page 20 he gives an illustration of the danger attending extraction of teeth, in this case resulting in the death of the patient through blood-poisoning.

The author confines his work mainly to diagnoses and therapeutics, varying this only, as in the several chapters on extraction, by giving the proper methods and precautionary measures, together with the preliminary and subsequent antiseptic care in the operation.

In the chapter on Extracting and Filling of Deciduous Teeth this account of teeth at birth is given: "A mother, of thirty-nine years, with two children, gave birth, after an interval of ten years, to a girl who at birth had eight teeth in the superior and inferior maxillæ. The teeth were cusped and firm, but were of a dirty yellow color."

Considerable space is given to Anæsthetics, Fractures, Transplantation, and Implantation.

The author's chapter on Pyorrhœa Alveolaris is neither full nor would the treatment be considered in this country as very efficient, but it has the merit of deeming the disease curable, which some on this side profess to doubt.

Space does not permit the reviewer to follow the pages of this excellent work. With the exception of Dr. Garretson's work, we have no book that treats as well as this the oral pathological conditions, and there is much here to be desired as the pages are read. It is thought that the author could improve this materially by entering more into detail as to treatment. It seems to the writer that this imperfection impairs its value as a text-book, for the average student demands explicit directions. It is one thing to have a formula for a prescription, and quite another to know the proper manner of using it.

The book, as a whole, must be considered as a valuable addition to the text-books of dentistry, and with American editing and additions would be worthy a place among the text-books in English for use in the schools. It would, however, require very much fuller treatment in many of the divisions than the author has seen fit to give to them.

